# Why many African countries may not achieve the 2022 COVID-19 vaccination coverage target

**DOI:** 10.1186/s41182-022-00407-6

**Published:** 2022-02-15

**Authors:** Israel Oluwaseyidayo Idris, Gabriel Omoniyi Ayeni, Yusuff Adebayo Adebisi

**Affiliations:** 1 Medair, Sudan Country Programme, Ecublens, Switzerland; 2 Department of Population Health, Faculty of Epidemiology and Population Health, London School of Hygiene and Tropical Medicine, London, UK; 3 Health Emergencies Programme (WHE) Department, World Health Organization WHO, Geneva, Switzerland; 4 Faculty of Pharmacy, University of Ibadan, Ibadan, Nigeria; 5 Global Health Focus, Kigali, Rwanda

**Keywords:** COVID-19, Vaccination, WHO target, Africa

## Abstract

COVID-19 continues to strain, stress, and stretch health systems globally. With the development of the COVID-19 vaccines, there are many issues still lurking behind the widespread coverage; one of which is COVID-19 vaccine nationalism and African countries are not exempted from these issues. This is evident in that many countries in the African region missed the earlier targets set by World Health Organization (WHO) for COVID-19 vaccination coverage. The WHO further set a target of 70% coverage of the COVID-19 vaccines for all countries by June 2022. In this article, we discuss the possible reasons why many African countries are struggling and may not achieve the COVID-19 vaccination target in 2022. With the fundamental issues facing COVID-19 vaccination ranging from nationalism to hesitancy, it is important that stakeholders continue to work harder to ensure that the continent is not left behind in the race to keep the world free and safe from the sting of the COVID-19 pandemic.

## To the Editor

Based on the current evidence derived from the clinical trials performed and ongoing data gathering from vaccine campaigns being conducted in several countries, achieving a protective level of herd immunity within populations against SARS-CoV-2 could be considered as one of the major goals of vaccinating against COVID-19 [1]. With the short shelf-life of COVID-19 vaccines, enormous vaccine wastage is inevitable especially in a low-resource setting where little or no resource capacity is available and private fund givers (donors) are backing out with response that COVID-19 vaccination programme is of low value for money due to the low utilization of the vaccine. This is even further complicated with the pervasive vaccine hesitancy in the African region [1]. The international goal to fully vaccinate 10% of every country’s population by the end of September 2021 and 40% of every country’s population by the end of 2021 was set at the World Health Assembly in May [2]. Even though almost 90% of high-income countries met the target, many African countries continue to struggle [3]. As at the end of December 2021, only 7 African countries with relatively smaller populations (Seychelles, Mauritius, Morocco, Tunisia, Comoros, Botswana, and Cape Verde) met the 40% target [3]. The WHO has set a further target of 70% coverage for all countries by June 2022, but this could also be missed across Africa [2]. In this commentary, we discuss the possible reasons why many African countries are struggling and may not achieve the COVID-19 vaccination target in 2022 and provide recommendations.

It has been identified that many African countries are incapacitated in tackling COVID-19-related mortality and morbidity due to paucity of well-equipped intensive care units for the management of severe COVID-19 cases [1]. In addition, the disease outbreak surveillance system in most countries does not cover the entire country and is structurally weak with long delays between an alert and confirmation of an outbreak [4]. This is absolutely associated with weak human resources for health capacity in the country, amongst other reasons [4]. The fundamentally weak health systems on the African continent are no doubt contributing to inadequate COVID-19 vaccination capacity and missing the ambitious target. Arguably, achieving a stretched but achievable target of COVID-19 vaccination coverage is the most hard-headed and realistic route to disrupt the outbreak and win the race against this virus in this context.

Controversially, the financing of COVID-19 vaccination programme could be considered to be the responsibility of national governments with the exception of nations in humanitarian crises, such as South Sudan, Ethiopia, Sudan, and Nigeria. It is important that this responsibility is shared between nations and donors. However, the issue of vaccine nationalism continues to undermine the COVID-19 vaccination efforts in Africa [5]. Waiving the long-debated intellectual property on COVID-19 vaccines is much-needed and will allow African countries to make their own vaccines. Although there are enough doses available from well-stocked countries to the low-resource countries to reach the world’s most vulnerable people, there is still a need for huge work and funding towards achieving a successful rollout. In fact, many African nations, for example, Sudan and Nigeria, experienced the burn-out of a specified large number of doses that had expired about a month of receival of shipment from donating nations [6]. While the African Union's African Vaccine Acquisition Trust has recommended at least more than 10 weeks of shelf-life on arrival of shipment for proper sub-national planning before countries accept donations from vaccine donating countries, it is necessary that recipients have a robust costing of its work plan to avoid a job half done in the implementation. This comprehensive work plan is recommended to be developed and implemented based on concrete evidence and best practices.

The current low COVID-19 immunization coverage reflects lack of commitment and quality of social mobilization, and in general, this is an exposure of the weak community engagement and planning capacity of many African health systems. It is not just the availability of the vaccines and vaccinations, but also the awareness and willingness to take it, that has been the major barrier in many resource-limited countries, precisely sub-Saharan African countries, to achieve the targeted coverage. If the pervasive vaccine hesitancy is not increasingly addressed, many African countries will still continue to miss the ambitious target.

While African nations continue to make efforts to design a feasible work plan to achieve the highest impact, the longer the delay in rolling out the vaccines, the greater the risk of additional challenges emerging, such as variants, hesitancy, operational gaps, or other threats. In this view, and in addition to establishing a stable supply of, and access to the ministries of health recommended COVID-19 vaccines, there is a need to improve the quality and quantity of communication to enhance informed COVID-19 vaccine uptake decision-making by the targeted population with the aim to achieve an increased utilization rate of the supplied vaccines. Beyond vaccination coverage, the concern about weak indicators to monitor and evaluate immunization progress should be addressed. Subsequent monitoring and evaluation of expenditures is equally essential. Furthermore, in-country contexts should be well factored into setting coverage goals and targets to make them more realistic and feasible.

Undoubtedly, there has been a stall and even reversal in other health agenda due to shifted attention to COVID-19 response [7, 8]. For example, non-communicable diseases (NCDs) have not received adequate attention despite its increased burden and associated implications, in African countries with high burden, such as Sudan, Egypt, and Nigeria among others [7]. This is even more worrisome, because people living with NCDs are at higher risk of severe COVID-19-related illness and death. All things being considered, sooner, there may be a reverse implication of other health agenda further complicating COVID-19 vaccination effort. Amid the pandemic, there has been a surge in outbreak of a number of vaccine-preventable diseases which in a way competing with the COVID-19 response efforts including vaccination [9, 10]. Substantial, prompt and carefully thought-out plans including funding and capacity expansion as well as solidarity among countries, which is currently lacking or limited, is required to stop the reverse trend, and recover the previous gains made from other health interventions, especially in Africa.

## Conclusion

Considering issues and challenges facing several African countries, the short timing in achieving the target is a concern, even though achieving it is desirable. While it is agreed that targets could be revised in the course of implementing programmes per context, the principle of goal setting demands that such should be done in a realistic time in the most appropriate context. Setting a goal of achieving at least 70% COVID-19 vaccination coverage by June 2022 in many African countries may be over-ambitious unless the driving factors change. African countries setting unrealistic targets is like digging deeper the foundation for repeated failure. Africans are not failures! African leaders and global partners including donors need to work harder to ensure the continent is not left behind in the race to keep the world free and safe from the sting of the COVID-19.

## Data Availability

Not applicable.

## Abbreviations

COVID-19: Coronavirus disease
NCDs: Non-communicable diseases
WHO: World Health Organization
